# Molecular Mechanisms in Seborrheic Dermatitis—Systematic Review

**DOI:** 10.3390/ijms27062799

**Published:** 2026-03-19

**Authors:** Sofiia Khimuk, Anastazja Andrusiewicz, Daniel Mijas, Danuta Nowicka

**Affiliations:** 1 Faculty of Medicine, Wroclaw Medical University, 50-367 Wrocław, Poland; 2 University Centre of General Dermatology and Oncodermatology, Faculty of Medicine, Wroclaw Medical University, 50-556 Wrocław, Poland

**Keywords:** seborrheic dermatitis, molecular mechanisms, Th1/Th17 immune signaling, oxidative stress, genetic and epigenetic regulation, epidermal barrier dysfunction

## Abstract

Seborrheic dermatitis (SD) is a chronic inflammatory skin disorder with a multifactorial pathogenesis involving immune dysregulation, oxidative stress, neuroendocrine signaling, and alterations of the epidermal barrier–lipid axis. Increasing molecular evidence indicates that SD is associated with both systemic and cutaneous abnormalities, including elevated β-endorphin levels, disturbed redox homeostasis, enhanced lipid peroxidation, dysregulated cytokine signaling, and genetic and epigenetic susceptibility factors. This systematic review was conducted in accordance with PRISMA guidelines. Comprehensive literature searches of PubMed, Scopus, and Web of Science identified eight studies that met the inclusion criteria. The included investigations comprised clinical case–control studies, genetic and epigenetic analyses, and multi-omics profiling of human blood and skin samples. Collectively, the findings demonstrate consistent systemic oxidative and neuroendocrine alterations alongside pronounced local immune activation characterized by Th1- and Th17-skewed responses, cytokine and stress-ligand upregulation, and activation of inflammatory signaling pathways. Genetic association signals and disease-specific microRNA profiles further implicate post-transcriptional regulation of immune and keratinocyte-related pathways in SD pathogenesis. Moreover, multi-omics studies revealed coordinated immune activation accompanied by impaired epidermal barrier function and altered lipid metabolism, supporting a dysregulated immune–barrier–lipid axis. Overall, SD emerges as a disorder driven by interconnected systemic and cutaneous molecular mechanisms. The identified pathways may represent promising directions for future biomarker research and targeted therapeutic development rather than established diagnostic or treatment strategies.

## 1. Introduction

Seborrheic dermatitis (SD) is a chronic, recurrent inflammatory skin disease. It is clinically characterized by pink or red, greasy, pruritic plaques that typically affect seborrheic areas of the body, including the nasolabial folds, eyebrows, chin, scalp, retroauricular regions, and presternal area [1].

The severity of SD varies and depends on multiple factors such as age, obesity, sex, alcohol consumption, smoking, psychological stress, seasonal changes, HIV infection, and neurological disorders, including Parkinson’s disease [2].

Although the etiopathogenesis of SD is not fully understood, three major factors are known to contribute to its development: skin barrier dysfunction, altered immune responses, and Malassezia overgrowth [3]. However, it has not been definitively established whether the relationship between Malassezia species and SD is causal or incidental [4].

In individuals with skin of color, SD may present with additional clinical features, including patches of hypopigmentation in typical seborrheic areas. A petaloid variant may also occur, characterized by pink, polycyclic, overlapping rings with subtle scaling. Pigmentary changes usually improve with appropriate treatment [5].

Epidemiological studies estimate that approximately 5% of the global population is affected by SD [6]. The disease occurs more frequently in men than in women and typically presents in infants within the first three months of life, as well as in adolescents and young adults. Its prevalence increases significantly in elderly individuals, particularly those over the age of 50 [7].

The prevalence of SD varies widely across geographic regions and populations. In rural India, the prevalence has been estimated at 0.25%, whereas in dermatology centers in Jamaica, it has been reported to be as high as 30.30%. Among imprisoned men in Italy, approximately 0.50% were affected, while in Italian geriatric dermatology clinics, the prevalence increased to 16.30% [8].

Immunocompromised individuals are particularly susceptible to SD, including organ transplant recipients, patients with lymphoma, and especially individuals infected with HIV. The prevalence of SD in HIV-positive patients reaches approximately 83% and is most commonly diagnosed when CD4+ T lymphocyte counts range between 200 and 500/mm^3^. HIV infection has also been associated with more severe manifestations of SD [4].

The Malassezia genus comprises 18 yeast species that colonize human skin, the gastrointestinal tract, and the surrounding environment. Malassezia species are considered opportunistic pathogens and have been implicated in several dermatological conditions. Their virulence factors include indole compounds, reactive oxygen species (ROS), hyphae formation, and biofilm production. Keratinocytes can recognize Malassezia through pattern recognition receptors, aryl hydrocarbon receptors responsive to indoles, and Toll-like receptors [9].

The pathogenesis of SD begins with lipid secretion by sebaceous glands onto the skin surface. These lipids are subsequently hydrolyzed by Malassezia species, resulting in the production of skin-irritating metabolites such as oleic acid, arachidonic acid, malassezin, and indole-3-carbaldehyde [7].

These metabolites interfere with keratinocyte differentiation and induce the production of pro-inflammatory cytokines, including IL-1α, IL-1β, IL-2, IL-4, IL-6, IL-10, IL-12, interferon-γ (IFN-γ), and tumor necrosis factor-α (TNF-α). Arachidonic acid is further metabolized into prostaglandins, which stimulate neutrophil recruitment and amplify the inflammatory response [10].

Protein thiol groups constitute one of the major antioxidative defense systems in the skin. Glutathione, a thiol compound synthesized primarily in the liver, is present in all cell types. Oxidation of thiols leads to the formation of disulfides, and the balance between these forms is known as thiol–disulfide homeostasis (TDH). Oxidative stress has been implicated in the inflammatory processes of SD, and studies have demonstrated elevated serum thiol levels in patients with SD, possibly reflecting a compensatory response to oxidative damage [11].

SD is a complex inflammatory skin disorder with a multifactorial pathogenesis involving immune, genetic, microbial, and neurogenic factors. Despite extensive research, many molecular mechanisms underlying SD remain incompletely understood, and available data are dispersed across numerous studies, limiting systematic comparison and analysis. This systematic review aims to compare and critically analyze studies investigating the molecular mechanisms of SD in adults, focusing on immune dysregulation, oxidative stress, cytokine signaling, microbiome-related pathogenic factors, genetic influences, and protein expression profiles in patients with SD compared to healthy controls. The ultimate goal is to provide an updated, evidence-based molecular perspective that may support the development of improved diagnostic and therapeutic strategies in the future.

## 2. Methods

The search was performed on 28 December 2025 in accordance with the Preferred Reporting Items for Systematic Reviews and Meta-Analyses (PRISMA) statement guidelines [12] using the databases PubMed, Scopus and Web of Science. The search strategy includes the following:

For PubMed: (“seborrheic dermatitis”[Title/Abstract] OR “seborrhoeic dermatitis”[Title/Abstract]) AND (pathogenesis[Title/Abstract] OR inflammation[Title/Abstract] OR immune*[Title/Abstract] OR cytokine*[Title/Abstract] OR “gene expression”[Title/Abstract] OR biomarker*[Title/Abstract] OR microbiome[Title/Abstract]) AND (“1 January 2015”[Date—Publication]: “31 December 2025”[Date—Publication]) AND English[Language]—272 documents.

For Scopus: TITLE-ABS-KEY (“seborrheic dermatitis” OR “seborrhoeic dermatitis” OR “seborrheic eczema” OR “seborrhoeic eczema”) AND TITLE-ABS-KEY (“molecular mechanism” OR “molecular mechanisms” OR “molecular pathogenesis” OR pathogenesis OR biomarkers OR “inflammatory mediators” OR “gene expression” OR cytokines OR cathelicidin OR LL-37 OR TLR2 OR “innate immunity” OR angiogenesis OR “matrix metalloproteinases” OR proteomics OR transcriptomics OR “skin microbiome”) AND (PUBYEAR > 2014 AND PUBYEAR < 2026) AND (LIMIT-TO (DOCTYPE, “ar”)) AND (LIMIT-TO (SUBJAREA, “MEDI”) OR LIMIT-TO (SUBJAREA, “BIOC”) OR LIMIT-TO (SUBJAREA, “IMMU”) OR LIMIT-TO (SUBJAREA, “ENVI”)) AND (LIMIT-TO (LANGUAGE, “English”))—391 documents.

For Web of Science: TS = (“seborrheic dermatitis” OR “seborrhoeic dermatitis” OR “seborrheic eczema” OR “seborrhoeic eczema”) AND TS = (“molecular mechanism” OR “molecular mechanisms” OR “molecular pathogenesis” OR pathogenesis OR biomarkers OR “inflammatory mediators” OR “gene expression” OR cytokines OR cathelicidin OR LL-37 OR TLR2 OR “innate immunity” OR angiogenesis OR “matrix metalloproteinases” OR proteomics OR transcriptomics OR “skin microbiome”) AND PY = (2015–2025) 205transcriptomics OR “skin microbiome”)—205 documents.

The records were independently screened by one reviewer at the title, abstract, and full-text levels, with verification by a second reviewer. Only studies that met all predefined eligibility criteria based on the PI(E)COS framework (“Population”, “Intervention”/“Exposure”, “Comparison”, “Outcomes”, and “Study design”) [13] were included, as presented in Table 1. A detailed flowchart of the selection process is provided in the Section 3.

Risk of bias was assessed using a set of checklists provided by the Joanna Briggs Institute (JBI) [14], for both case–control studies and cross-sectional studies. For case–control studies, the assessment included criteria related to the appropriateness of case and control selection, comparability of groups, validity and reliability of exposure measurement, identification and management of confounding factors, and adequacy of statistical analysis. For cross-sectional studies, the checklist addressed clearly defined inclusion criteria, validity and reliability of exposure and outcome measurements, identification of confounders, and appropriateness of analytical methods.

## 3. Results and Discussion

**Figure 1:**
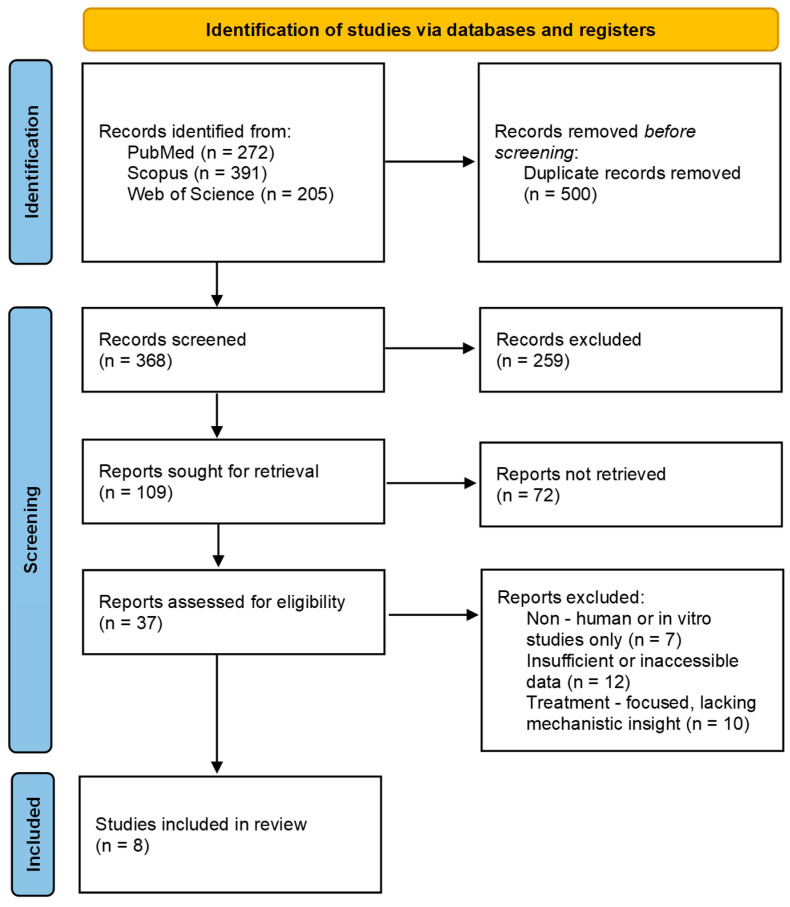
PRISMA flowchart of selected studies.

In the search, we identified 868 records (272 in PubMed; 391 in Scopus; 205 in Web of Science). Out of these, 500 duplicate records were removed. 368 titles and abstracts were screened, and out of these, 259 were excluded. The remaining 109 articles were sought for retrieval, and 37 were assessed for eligibility. Eight articles met the inclusion criteria and were summarized in this review. The selection process is presented in the PRISMA flowchart (Figure 1).

The eight studies included in this review, published between 2018 and 2025, were conducted across multiple geographical regions, including Turkey (1 study), China (2 studies), Ukraine (1 study), Bangladesh (1 study), the Netherlands (1 study), Korea (1 study) and the USA (1 study). Collectively, the studies evaluated approximately 867 patients with SD and 3638 controls, with the disproportionately large number of controls primarily attributable to a population-based genetic cohort. The study designs comprised five observational case–control studies, one observational case–control study, genetic association study, one prospective cohort study, and one observational cross-sectional study. Sample sizes ranged from small mechanistic studies enrolling 4–16 patients to large-scale genomic analyses involving over 4000 participants, thereby providing complementary systemic, cutaneous, and molecular perspectives on SD pathophysiology.

The characteristics of the included studies, including participants, molecular focus and principal findings, are summarized in Table 2.

### 3.1. Neuroendocrine and Oxidative Regulatory Mechanisms in SD

Three of the eight included studies focused on systemic neuroendocrine and redox-related alterations in SD, highlighting β-endorphin signaling, thiol/disulfide imbalance, and increased systemic oxidative stress. These findings suggest that dysregulation of redox homeostasis and neuroendocrine regulatory mechanisms may contribute to disease susceptibility in SD.

Vysochanska et al. [15] examined plasma β-endorphin levels in 62 patients with SD (35 males and 27 females; mean age 32.9 ± 1.55 years) and 26 healthy controls (10 males and 16 females; mean age 34.88 ± 1.79 years). β-endorphin levels were significantly higher in SD patients compared to controls (35.5 vs. 22 pg/mL, *p* < 0.001) and positively correlated with disease severity (SEDASI score, r = 0.42, *p* < 0.001) and pruritus intensity (r = 0.332, *p* = 0.009). β-endorphin levels increased progressively from mild to very severe SD, whereas no significant associations with age or sex were observed. The findings suggest that elevated β-endorphin expression contributes to SD pathogenesis through modulation of immune responses, sebaceous gland activity, and pruritus, highlighting a potential target for personalized therapeutic strategies

Emre et al. [16] examined thiol/disulfide homeostasis in 70 patients with SD (47 males and 23 females; mean age 31.87 ± 11.13 years) and 61 healthy controls (34 males and 27 females; mean age 35.82 ± 13.04 years). Native and total thiol levels were significantly higher in SD patients compared to controls (both *p* < 0.001), while disulfide levels were slightly lower and not statistically significant (*p* = 0.821). Age and age at disease onset were negatively correlated with native and total thiol levels, whereas disease duration and severity were positively associated with thiol/disulfide levels. These findings suggest that elevated thiol levels may reflect a compensatory response to oxidative stress and could contribute to increased proliferation of SD lesions, providing novel insight into redox-related mechanisms in SD pathogenesis.

Jahan et al. [17] conducted a case–control study in 75 patients with SD (43 males and 32 females; mean age 27.13 ± 1.61 years) and 76 age- and sex-matched healthy controls (41 males and 35 females; mean age 26.30 ± 0.92 years) to investigate systemic oxidative and immunological alterations. Patients with SD exhibited significantly elevated serum levels of malondialdehyde together with increased concentrations of multiple trace elements, indicating enhanced lipid peroxidation and oxidative stress, while reduced immunoglobulin levels (IgA, IgG, IgM) suggested impaired systemic immune regulation. Additionally, disease-specific inter-elemental correlations, including altered copper–calcium and zinc–calcium relationships, point to disrupted redox homeostasis. Collectively, these findings support the involvement of systemic oxidative regulatory mechanisms in the pathophysiology of SD.

### 3.2. Genetic, Epigenetic, and Multi-Omics Architecture

Of the eight included studies, two addressed the genetic and epigenetic architecture of SD. These studies identified disease-associated microRNA expression patterns and genetic susceptibility signals, supporting a molecular regulatory contribution to SD pathogenesis.

Sanders et al. [19] conducted a candidate gene approach and pilot genome-wide association study within the Rotterdam Study cohort, including 4050 participants, of whom 609 were diagnosed with SD (368 males and 241 females; mean age 68.94 ± 9.3 years) and 3441 served as controls (1437 males and 2004 females; mean age 67.97 ± 9.6 years). The estimated heritability of SD was approximately 14%, and two significant genome-wide loci were identified, including rs58331610 within the MAST4 gene and rs16944244 in an intergenic regulatory region. Additional suggestive associations involved immune- and barrier-related genes such as LCE3, IL12B, and MICB, while bioinformatic analyses indicated regulatory roles of inflammatory transcription factors and chromatin remodeling, supporting a genetic contribution to immune regulation and epidermal integrity in SD.

Kim et al. [20] performed a paired lesion–non-lesion microarray study to investigate microRNA dysregulation in SD, analyzing scalp biopsy samples from five elderly male patients (5 males and 0 females; mean age 74.6 years, range 72–77 years). Eight differentially expressed miRNAs were identified in lesional skin, including upregulation of miR-20a-5p, miR-106b-5p, miR-191-5p, miR-127-3p, miR-342-3p, and miR-6824-5p and downregulation of miR-6831-5p and miR-7107-5p, with findings validated by qRT-PCR. Target gene and pathway analyses revealed enrichment of immune signaling, cytokine pathways, cell cycle regulation, and apoptosis, indicating that altered miRNA-mediated post-transcriptional regulation contributes to immune activation and aberrant keratinocyte proliferation in SD.

### 3.3. Immune–Barrier–Lipid Interactions in SD

Three studies addressed the complex interplay between immune responses, epidermal barrier integrity, and lipid dysregulation in SD, integrating transcriptomic, proteomic, and multi-omics approaches to elucidate convergent pathogenic pathways.

Ungar et al. [21] performed facial tape-strip RNA sequencing in 26 adult patients with SD (19 males and 7 females; mean age 41.2 ± 11.4 years) and 18 healthy controls (12 males and 6 females; mean age 38.6 ± 14.4 years). Transcriptomic analysis identified 1037 differentially expressed genes, including 556 upregulated and 481 downregulated genes in SD compared with controls. Lesional skin demonstrated a predominant Th17/Th22 immune signature, with increased expression of IL-23/Th17–related cytokines and innate immune markers, together with downregulation of lipid metabolism and epidermal barrier–associated genes, indicating coordinated immune activation and barrier dysfunction in SD.

Shen et al. [18] analyzed lesional scalp skin from 16 patients with SD (9 males and 7 females; mean age 45.4 ± 11.6 years) and 12 healthy controls (7 males and 5 females; mean age 40.8 ± 14.4 years) using proteomic profiling. Sixteen proteins were differentially expressed in lesional skin, with upregulation of Th1-related cytokines (IL-18, IL-18R1), chemokines, and T-cell activation molecules (CCL19, CD6, CD244, CASP-8, TNFSF14, PD-L1, CD5, CD40), while IL-1α was downregulated. Pathway analysis revealed Th1-skewed immune activation, cytokine-storm signaling, and CGAS–STING pathway engagement, alongside alterations in lipid metabolism–related pathways, supporting a link between immune activation and barrier–lipid dysregulation in SD.

Yang et al. [22] performed a multi-omics analysis of human scalp biopsy samples, including lesional and non-lesional skin from patients with SD and scalp psoriasis, as well as healthy scalp controls, with four samples analyzed per condition (age range 20–54 years; sex and mean age not reported). RNA sequencing identified differentially expressed genes associated with immune activation, epidermal barrier dysfunction, and lipid metabolism. Lesional SD showed enrichment of chemokine signaling and arachidonic acid metabolism, whereas progression toward scalp psoriasis was characterized by increased activation of IL-17, RIG-I–like receptor, and JAK–STAT signaling pathways. Network analysis identified TGM1 and IL36RN as key hub genes expressed in granular keratinocytes, further supporting a dysregulated immune–barrier–lipid axis in SD.

**Figure 2:**
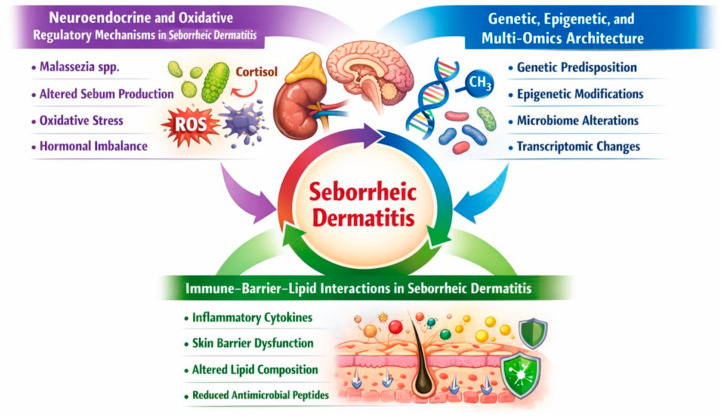
Molecular mechanisms underlying the pathogenesis of seborrheic dermatitis.

To illustrate the molecular pathogenesis of SD, the key mechanisms implicated in the development and persistence of this chronic inflammatory dermatosis are summarized in Figure 2.

### 3.4. Quality Assessment of the Included Studies

Seven studies had a case–control design, while one was cross-sectional. Among the case–control studies, quality scores ranged from 4 to 10 on a 10-point scale. The cross-sectional study received a score of 8 out of 10. Details are shown in Table 3.

The main methodological limitation was related to the reporting of diagnostic procedures to confirm the diagnosis and control matching. In several studies, participants were already under the supervision of dermatology clinics due to SD, and the diagnosis was based primarily on medical records. For this reason, no standardized verification of the diagnosis was performed prior to enrollment, and historical data such as skin biopsy results were not collected to exclude other dermatologic conditions with overlapping clinical features.

A second limitation involved limited reporting on confounding variables and detailed clinical data. Most studies were designed to investigate molecular mechanisms rather than correlations with clinical severity or phenotype and therefore did not comprehensively assess or adjust for potential confounders. Consequently, conventional quality appraisal checklists may not fully capture the methodological strengths and limitations specific to mechanistic molecular studies.

### 3.5. Discussion

This systematic review integrates current evidence on the molecular mechanisms underlying SD, highlighting the interplay between systemic neuroendocrine and oxidative disturbances, localized immune activation, and dysregulation of epidermal barrier and lipid metabolism. Collectively, the reviewed studies support the concept of SD as a multifactorial inflammatory disorder driven by convergent systemic and cutaneous molecular pathways rather than a single etiological factor.

Systemic neuroendocrine and redox-related alterations emerged as important upstream contributors to disease susceptibility. Elevated plasma β-endorphin levels were consistently associated with disease severity and pruritus intensity, suggesting that neuroendocrine signaling may modulate immune responses and sebaceous gland activity, thereby influencing SD pathogenesis [15]. Keratinocytes, sebaceous glands, and nerve endings express μ-opioid receptors and can be activated by circulating or locally produced β-endorphin. Prolonged exposure to high β-endorphin levels reduces receptor sensitivity, potentially contributing to chronic inflammation. Persistently elevated BE levels may suppress T-cell maturation and IgG production; stimulate sebaceous lipid secretion; promote pruritus, angiogenesis, and hyperkeratosis; and enhance NK cell activity, thereby sustaining inflammatory processes in the skin [23,24]. In parallel, multiple studies demonstrated altered oxidative balance in SD patients, characterized by increased lipid peroxidation, disrupted trace element homeostasis, and changes in thiol/disulfide status, indicating sustained systemic oxidative stress [16,17]. These findings imply that redox dysregulation may both reflect and amplify inflammatory processes, potentially promoting keratinocyte proliferation and lesion persistence.

At the genetic and epigenetic level, evidence supports a heritable and post-transcriptionally regulated component of SD. Population-based analyses revealed a modest but measurable heritability and identified genome-wide significant loci within genes involved in immune regulation and epidermal barrier integrity, reinforcing the contribution of host genetic susceptibility [19]. Complementary epigenetic profiling identified distinct microRNA expression patterns in lesional skin, with predicted targets enriched in immune signaling, cytokine pathways, apoptosis, and cell-cycle regulation, indicating that miRNA-mediated post-transcriptional control contributes to sustained immune activation and abnormal keratinocyte behavior in SD [20].

Local cutaneous immune activation represents a central pathogenic mechanism linking systemic susceptibility to clinical disease expression. Proteomic profiling of lesional scalp skin revealed a predominantly Th1-skewed inflammatory signature, characterized by upregulation of IL-18, IL-18R1, and multiple T-cell activation and co-stimulatory molecules, together with activation of cytokine-storm–associated and CGAS–STING signaling pathways [18]. The relative absence of Th2-associated mediators suggests a polarized immune response distinct from classical allergic inflammation and aligns with an innate immune–driven disease model.

Transcriptomic and multi-omics studies further elucidated the coordinated dysregulation of immune responses, epidermal barrier function, and lipid metabolism. Tape-strip RNA sequencing demonstrated robust Th17/Th22 immune activation alongside downregulation of genes involved in lipid metabolism and barrier formation, indicating a tightly coupled immune–barrier axis in SD lesions [21]. Multi-omics scalp analyses confirmed enrichment of chemokine signaling and arachidonic acid metabolism in SD, while progression toward psoriatic features was associated with increased activation of IL-17, RIG-I–like receptors, and JAK–STAT signaling pathways. Network-based approaches identified keratinocyte-expressed hub genes involved in cornification and inflammatory regulation, underscoring the central role of the immune–barrier–lipid axis in disease pathophysiology [22].

Taken together, the reviewed evidence supports a unifying pathogenic framework in which systemic neuroendocrine and oxidative disturbances, shaped by genetic and epigenetic susceptibility, prime the skin for exaggerated immune responses. These responses—dominated by Th1 and Th17 pathways—drive epidermal barrier impairment and lipid metabolic alterations, creating a self-perpetuating inflammatory microenvironment characteristic of SD. Key molecular pathways identified across studies, including CGAS–STING signaling, IL-17-mediated immunity, and keratinocyte regulatory networks, represent promising targets for future biomarker development and mechanism-based therapeutic strategies.

Despite advances in molecular profiling, several important gaps remain in the current understanding of SD. Most included studies provided limited clinical characterization, with insufficient patient stratification according to disease subtype, severity, age, or comorbid conditions, some of which may overlap mechanistically with SD. Confounding factors, particularly those influencing inflammatory signaling and gene expression, were inconsistently addressed. Moreover, associations between molecular alterations and clinical parameters such as disease activity, remission status, or severity scores were rarely explored. To date, available studies have been small and predominantly cross-sectional, capturing molecular signatures at a single time point. The absence of longitudinal investigations limits insight into dynamic changes in biomarker expression and pathway activity over the disease course, as well as their potential utility for disease monitoring. Future research should prioritize well-characterized, stratified cohorts; standardized reporting of clinical variables; adjustment for relevant confounders; and longitudinal multi-omics approaches to better define causal mechanisms and clinically meaningful biomarkers.

## 4. Conclusions

This systematic review demonstrates that SD is a molecularly complex inflammatory disorder characterized by the convergence of systemic neuroendocrine and oxidative dysregulation with localized immune, barrier, and lipid abnormalities. Evidence from available human studies suggests that Th1- and Th17-skewed immune signaling, oxidative stress imbalance, and post-transcriptional and genetic regulatory mechanisms may contribute to disease susceptibility and lesion persistence. Genetic predisposition and miRNA-mediated regulation appear to modulate inflammatory and keratinocyte pathways, while multi-omics analyses point toward disruption of the immune–barrier–lipid axis as a potentially central pathogenic feature.

Together, these findings support a multi-layered molecular model of SD; however, given the limited number and heterogeneity of included studies, these mechanisms should be interpreted cautiously. The identified pathways may represent promising directions for future biomarker research and targeted therapeutic development rather than established diagnostic or treatment strategies.

## Figures and Tables

**Table 1:** Eligibility criteria.

Parameter	Inclusion Criteria	Exclusion Criteria
Population	Human participants diagnosed with SD (any clinical subtype: mild, moderate, severe). Adults (≥18 years).	Participants without a confirmed diagnosis of SD. Studies including only pediatric populations (<18 years).
Intervention/Exposure	Assessment of molecular biomarkers, signaling pathways, or gene/protein expression related to the pathogenesis of SD.	Research focused primarily on therapeutic interventions without investigation of molecular mechanisms. Studies assessing only the skin or gut microbiome composition without molecular pathway analysis.
Comparison	Healthy controls; if unavailable, non-lesional skin or internal patient comparisons.	Studies without any valid comparator (no healthy controls, no non-lesional samples, no pre–post design, and no reference range).
Outcomes	Quantitative or qualitative data on molecular mechanisms (e.g., cytokines, oxidative stress markers, transcription factors, immune cell profiles, skin barrier proteins).	Lack of quantitative or qualitative data on molecular biomarkers, signaling pathways, or gene/protein expression relevant to SD. Studies reporting only clinical outcomes without molecular analysis.
Study design	Original research articles (case–control, cross-sectional, cohort, experimental studies). Studies providing data from human samples, even if combined with complementary in vitro or animal experiments.	Full version of the document not available Non-English language publications. Published before 2015 Literature reviews, editorials, commentaries, letters to the editor, and case reports.

SD, seborrheic dermatitis.

**Table 2:** Characteristics of included studies.

Author	Study Design	Participants	Biological Material	Molecular Mechanisms Studied
Vysochanska [15] 2023 Ukraine	observational case–control study	SD patients (*n* = 62) Controls (*n* = 26)	blood	β-endorphin-mediated immune modulation, pruritus-related signaling
Emre [16] 2020 Turkey	observational case–control study	SD patients (*n* = 70) Controls (*n* = 61)	blood	oxidative stress regulation, thiol/disulfide redox balance
Jahan [17] 2021 Bangladesh	observational case–control study	SD patients (*n* = 75) Controls (*n* = 76)	blood	systemic oxidative stress, immune dysregulation
Shen [18] 2025 China	observational case–control study	SD patients (*n* = 16) Controls (*n* = 12)	punch biopsy from an active inflammatory lesion	T-cell/lymphocyte activation, the cytokine storm signaling pathway and the cGAS–STING signaling pathway
Sanders [19] 2018 Netherlands	cross-sectional study	SD patients (*n* = 609) Controls (*n* = 3441)	blood	genetic susceptibility, immune and barrier regulation
Kim [20] 2022 Korea	case–control study	SD patients (*n* = 5) Controls (*n* = 0)	punch biopsy from the lesional area and the non-lesional region area	microRNA dysregulation, immune signaling, keratinocyte proliferation
Ungar [21] 2025 USA	observational case–control study	SD patients (*n* = 26) Controls (*n* = 18)	tissue samples collected by applying tape-strips to facial lesions	Th17/Th22 activation, barrier dysfunction, lipid metabolism
Yang [22] 2025 China	observational case–control study, genetic association study	SD (lesional *n* = 4; non-lesional *n* = 4); scalp psoriasis (lesional *n* = 4; non-lesional *n* = 4); healthy controls (*n* = 4)	punch biopsy from non-inflammatory melanocytic nevi, and lesional and adjacent normal areas	IL-17 and JAK–STAT signaling, barrier gene TGM1, lipid metabolism

β-endorphin—beta-endorphin, cGAS–STING—cyclic GMP–AMP synthase–stimulator of interferon genes, IL-17—interleukin 17, JAK–STAT—Janus kinase–signal transducer and activator of transcription, microRNA—micro ribonucleic acid, SD—Seborrheic dermatitis, TGM1—transglutaminase 1, Th17—T helper 17 cells, Th22—T helper 22 cells.

**Table 3:** Risk-of-bias evaluation of the included studies.

Case–Control Studies	Emre	Jahan	Kim	Shen	Ungar	Vysochanska	Yang
Were the groups comparable other than presence of disease in cases or absence of disease in controls?	Yes	Yes	Yes	Yeas	Yes	NR	NR
Were cases and controls matched appropriately?	Yes	Yes	Yes	Yes	NR	NR	NR
Were the same criteria used for identification of cases and controls?	Yes	Yes	NA	NR	Yes	Yes	Yes
Was exposure measured in a standard, valid and reliable way?	Yes	Yes	NR	NR	NR	Yes	NR
Was exposure measured in the same way for cases and controls?	Yes	Yes	Yes	Yes	Yes	Yes	Yes
Were confounding factors identified?	Yes	NR	Yes	NR	NR	NR	NR
Were strategies to deal with confounding factors stated?	Yes	NR	Yes	NR	NR	NR	NR
Were outcomes assessed in a standard, valid and reliable way for cases and controls?	Yes	Yes	Yes	Yes	Yes	Yes	Yes
Was the exposure period of interest long enough to be meaningful?	Yes	Yes	Yes	NR	Yes	NR	NR
Was appropriate statistical analysis used?	Yes	Yes	Yes	Yes	Yes	Yes	Yes
Total	10/10	8/10	8/10	5/10	6/10	5/10	4/10
**Cross-Sectional Studies**				**Sanders**			
Were the criteria for inclusion in the sample clearly defined?				Yes			
Were the study subjects and the setting described in detail?				Yes			
Was the exposure measured in a valid and reliable way?				Yes			
Were objective, standard criteria used for measurement of the condition?				Yes			
Were confounding factors identified?				NR			
Were strategies to deal with confounding factors stated?				NR			
Were the outcomes measured in a valid and reliable way?				Yes			
Was appropriate statistical analysis used?				Yes			
Total				6/8			

## Data Availability

No new data were created or analyzed in this study. Data sharing is not applicable to this article.

## Abbreviations

The following abbreviations are used in this manuscript:

CASP-8	Caspase 8
CCL19	C–C motif chemokine ligand 19
CD4+	Cluster of differentiation 4–positive
cGAS	Cyclic GMP–AMP synthase
cGAS–STING	Cyclic GMP–AMP synthase–stimulator of interferon genes pathway
GWAS	Genome-wide association study
HIV	Human immunodeficiency virus
IFN-γ	Interferon gamma
IgA, G, M	Immunoglobulin A, G, M
IL	Interleukin
IL-1α	Interleukin 1 alpha
IL-1β	Interleukin 1 beta
IL-2, 4, 6, 10, 12, 17, 18, 23	Interleukins 2, 4, 6, 10, 12, 17, 18, 23
IL-18R1	Interleukin 18 receptor 1
IL36RN	Interleukin 36 receptor antagonist gene
JAK–STAT	Janus kinase–signal transducer and activator of transcription pathway
LCE3	Late cornified envelope protein 3
MAST4	Microtubule-associated serine/threonine kinase 4
MAPK	Mitogen-activated protein kinase
MDA	Malondialdehyde
MICB	MHC class I polypeptide-related sequence B
miRNA	MicroRNA
NF-κB	Nuclear factor kappa B
PD-L1	Programmed death-ligand 1
qRT-PCR	Quantitative real-time polymerase chain reaction
RIG-I	Retinoic acid–inducible gene I
RNA	Ribonucleic acid
RNA-seq	RNA sequencing
ROS	Reactive oxygen species
SD	Seborrheic dermatitis
SEDASI	Seborrheic Dermatitis Area and Severity Index
SSD	Scalp seborrheic dermatitis
STAT3	Signal transducer and activator of transcription 3
STING	Stimulator of interferon genes
TDH	Thiol–disulfide homeostasis
TGM1	Transglutaminase 1
Th	T helper cells
Th 1, 17, 22	Type 1, 17, 22 T helper cells
TLR	Toll-like receptor
TLR 2, 4, 7	Toll-like receptor 2, 4, 7
TNF-α	Tumor necrosis factor alpha
TNFSF14	Tumor necrosis factor superfamily member 14
